# A Report on Typhoid Fever Made to the Scott County Medical Society

**Published:** 1850-10

**Authors:** W. L. Sutton

**Affiliations:** Georgetown, Ky.


### WESTERN JOURNAL

OF

### MEDICINE AND SURGERY.

OCTOBER, 1850.

ART. I.—*A Report on Typhoid Fever made to the Scott County Medical Society*. By W. L. SUTTON, M. D., of Georgetown, Ky.

(CONTINUED.)

### CHAPTER IV.

### ARTICLE 1.

#### DURATION AND PROGNOSIS.

There is much difficulty in determining the duration of many cases of fever. Very frequently the advent is so insidious that its presence is not noticed until the patient becomes seriously ill, and when the physician sees him, he is not able to give any satisfactory account of his previous state or feelings. Again, for several days, the change for the better cannot be appreciated so as to say with any assurance on what day it commenced. Add to this, that in a given proportion of cases, there is an apparent but probably no real amendment, which is succeeded by an aggravation of symptoms and fatal termination.

It seems to me that the time at which convalescence is decided by Dr. Jackson to have commenced, viz.: "When the patient is able to take a moderate allowance of solid food, the febrile symptoms having subsided for at least two or three days previous to this period," is somewhat objectionable. I think it should be dated from the time when amendment is decided. It seems to me too, that it is not safe to allow solid food for several days, say a week after amendment is evident.

Keeping these obstacles in mind, I will state that on the 6th of July, 1846, Dick, a negro boy eighteen years old sickened, not being known to be ill before; he died on the 12th. Mrs. S. sickened on the 14th of July, having been a good deal indisposed for two weeks, and having lost much flesh; she died on the 20th. These, I think are the shortest courses of fatal cases, which have fallen under my observation. Whether Dick had been indisposed, but chose to conceal it, I do not know. His attack seemed to be sudden and exceedingly severe. I saw him on the second day of his disease, when his pulse was one hundred and thirty, great distention and tenderness of abdomen, profuse diarrhea, and delirium. I was visiting the house daily when Mrs. S. gave up; she had no fever or diarrhea before her attack. In fact I had made some laxative pills for her the day before her attack, of which however she had not taken any.

J. A. R. aged eight had been complaining of debility two weeks, sleepy, and heavy; was taken April 30, 1847, at night; chilly, afterwards feverish. May 1st—vomited some bile, soon after which I saw him; face red, eyes watery and injected; tongue whitish with red papillæ, lips vermilion; no diarrhea, slight meteorism; gurgling at the right iliac region; pulse one hundred and twenty. ℞. Ipecac. grs. xv. Got very little of it on account of vomiting every time it was put to his lips—threw up a little bile, no phlegm. Within a few minutes after taking ipecac. had a liquid fetid cider stool, and another in fifteen or twenty minutes. ℞. Cal. mass. hydrarg, aa. grs.; iv. pulv. Dover's p. grs. iii. nocte. 2d. Had no stool since I left until this morning, when he had three, much as those of yesterday, pulse 110, otherwise as yesterday. ℞. No med. 3d. Appeared so much relieved that I prescribed nothing and ceased attendance. 7th. Saw him on the farm where his father was at work. This may not be considered a case of typhoid fever, or it may be considered one which had remained latent. However it may appear to others, I certainly, at the time, thought it a well marked case; one that was likely to give me trouble. With the exception of a few such cases as the preceding, I have found those presenting such symptoms go on for two or three weeks.

I saw John Sutphin on the 20th of June, 1846, he having been sick three weeks; he died 12th July, making six weeks illness. This is the longest case that I have attended regularly, and this only during the latter half of his illness. I have seen one case however who was confined twelve weeks or upwards, a large portion of that time being in a very precarious situation. Such may be considered the extremes of cases seen by me.

In general there is very gradual aggravation of symptoms for six or eight days; then there are from four to eight days, during which no appreciable change takes place. Then there is a very gradual amendment for five or six days more, and then a more rapid improvement.

It would seem that the severity of the attack has very little to do with its duration. How mild soever it may be, it is apt to last two or three weeks. For instance I was myself confined during the last summer, (1847,) for five weeks with, as I think, typhoid fever. There was scarcely a day during the time, when I did not feel as if I were well enough to ride twenty miles; yet I could not sit up with comfort half an hour. After the first few days, I scarcely had any appreciable evidence of disease; and my brethren who visited me frequently, saw no indication for medicine. One of them remarked one day rather dryly, "that if he were lying in bed so long without more manifestation of disease, he should think he had the hypo." After the first few days my pulse ranged from sixty to eighty-five; yet during the fifth week, owing to a very slight indiscretion in eating, and sitting up long enough to write a letter of half a page, my pulse rose up to one hundred and twenty.

Circumstances like this go farther to support the idea of a specific character of this disease than any thing else that I know of.

---

### ARTICLE 2.

### PROGNOSIS.

The prognosis of bad cases is made out with considerable difficulty. We may have some very grave symptoms united with others of better omen, viz.: We may have a frequent pulse, perhaps joined with a dry, smooth tongue, but the mind may be clear; the expression of countenance good; the bowels not much vexed with diarrhea, little distention or tenderness of the abdomen; no subsultus tendinum. Again, the pulse may be quick with considerable distension and tenderness of the abdomen; but the mouth moist, little diarrhea; soft skin, no delirium, &c.

Great frequency of pulse, long continued profuse diarrhea, quick respiration, especially if performed irregularly, dry, black tongue, profuse hemorrhage at the latter stages of the disease; subsultus tendinum, low muttering delirium; loud raving; great prostration; discharges of flatus; stranguary, suppression of urine, restlessness, and an impression of being well when very grave symptoms are present, are among the symptoms indicating great danger. If I were to select three symptoms indicating much peril, I would take a frequent weak pulse, profuse and continued diarrhea, and a dry, black tongue. Whatever other symptoms may be present, I hold that a combination of these is of evil import. I do not now remember a case of doubtful aspect, in which one or more of these symptoms were not present; and the converse is true. I do not remember a case in which the three were united which was not a grave case. As one or more of these three, united with other symptoms enumerated as indicating danger, are present so will the danger of the case be.

We must remember however, that there is no disease in which there is more reason to hope, against hope. Thus in a case in which there had been continued diarrhea, with quick pulse, and great muscular prostration, profuse epistaxis came on in the last stage, and death seemed to be averted only by plugging the nostrils; yet recovery was at last rapidly effected. It may be proper to notice a case which I saw in 1849. A young gentleman had been very low for more than a week. Among the grave symptoms was free hemorrhage from the bowels. After one of these discharges he sank very much, pulse became very frequent and too weak to be counted, and it seemed as if death must speedily ensue. He seemed at least as low, if not lower than the last case. Yet he recovered notwithstanding the continuance of hemorrhage from the bowels for twelve or fifteen hours after.

Again, in a case of extreme prostration, profuse diarrhea, delirium, and where in truth the patient was abandoned by his physician, the pulse however remaining tolerable steady, recovery took place. Thus it is that in all cases we must lean to the side of hope. On the other hand we must not forget that there is another side to the picture. In a few cases in which the disease has apparently been mild, when in fact no very grave symptom has shown itself, when the convalescence is considered as already set in, a perforation of the bowel may take place, and death speedily close the scene. I have seen a case in which for four weeks the pulse ranged from sixty to eighty; the bowels never much troubled; the tongue nearly natural; no undue heat of skin; indeed no bad symptom occurred, if we except a moderate but obstinate pain in the occiput. During all this time the patient set up more or less every day, generally in a room adjoining his bed-room, yet at the expiration of this time he began to become deaf, his pulse slowly increased in frequency, his tongue became gradually coated and dry; presently delirium set in, and during the sixth week he died.

Again, a case may be severe, but improvement may appear to have commenced and continued for two or three days, when an aggravation of symptoms may take place, and the patient steadily grow worse, until a fatal termination ensues. This aggravation may occur without any cause apparent to the physician, or nurse. It is frequently charged upon improper indulgence. If this last has been the cause, we may hope by diligence and attention to remedy the evil, but if it is not, the prospect is truly gloomy, as I do not remember a case of recovery from this state.

I have found profuse hemorrhage in the last stage, much less fatal than I was prepared to expect from reading or reflection. I remember but ten cases in which it occurred. In two only did death follow.* In two cases where I was satisfied a perforation of the bowel had taken place, and when the patient evidently was to live but a few hours, each was impressed firmly with the idea that nothing was the matter.

I have found stranguary and suppression of urine, both to be symptoms of evil import. The patient, from whom it was necessary to draw the urine daily for six weeks, was the only case of recovery in which either symptom was observed.

The case above mentioned, in which a general rigidity of the muscles occurred, recovered. I ought perhaps to state that there was no permanent rigidity, but it came on when the body was to be moved. It continued to recur for thirty-six or forty-eight hours.

---

### CHAPTER V.

### APPEARANCE ON DISSECTION.

Dissections of patients dying of this affection, have not been made, in this vicinity at least, at all commensurate with the gravity of the disease, or the interest of the profession. Doubtless, dissections have been made—but in 1841, I made my first post mortem examination; and at that time, I was not aware that any proof of identity between this disease and the typhoid fever of France and New England existed, save in the symptoms during life. Indeed, I made a special inquiry of Dr. Holland, at the time he was called to see my first lot of cases, if any dis-

*Since writing the above I have seen several other cases but no death.

sections had been made in Lexington or its vicinity, to determine the point, and he told me that he was not aware of any. I have made but three autopsies; two patients having died about the fifteenth day, the other on the thirty-first. I have also a cast of a portion of the ilium, taken by my son from a patient who died at the end of the third week.

A. Stubbs, aged twenty-three, had been sick fifteen days, during which time great general uneasiness, considerable pain in the abdomen and throat, excessively dry, cracked tongue, and coolness of the extremities, were the most marked symptoms. Meteorism, or diarrhea was never considerable; pulse for a week before death one hundred and fifteen to one hundred and twenty-five; no rose spots; no sudamina until the day of his death, when a crop appeared upon his face and neck. Died March 15, 1846.

*Autopsy twenty-three hours after death.*—Muscular contraction very rigid; abdomen not distended; the skin as hard to cut as parchment; [a blister had been applied on the abdomen on the 5th;] the omentum very much injected and extended down to the pubis; the bowels, when exposed by raising the omentum, appeared considerably injected, their calibre diminished. *Liver*, apparently healthy. *Stomach*, externally of good appearance, internally considerably injected below the middle depression, and empty, except that the surface was covered with good looking bile. *The intestines* throughout were lined with the same bilious matter, otherwise empty; the small intestines red internally throughout their course; near the ilio-cecal valve they were more highly colored externally, and internally presented elliptical plates distinctly elevated and some of them ulcerated in numerous distinct minute points; solitary glands distinctly thickened, and some of them ulcerated; the mucus membrane considerably injected, having veins distended with dark blood interspersed. *Colon*, externally many blood vessels; the calibre very much diminished, cord-like, the lateral pouches being nearly obliterated. The spleen, pancreas, and kidneys, not examined; the thorax not opened. In fact we had to make the examination surreptitiously, and in the house where there were more lying very ill, and, of course, imperfectly.

2d. Ben, aged twenty-five, had been sick about two weeks; profuse diarrhea, mostly of a dirty black green. At first considerable pain in abdomen, subsequently but little; considerable tympanites; tongue furred, whitish; pulse from seventy to eighty, never became frequent. During his illness he vomited a dark green bile, but not enough for it to be considered a prominent symptom. There had been great muscular weakness; but Dr. C., who was kind enough to invite me to witness the autopsy, informed me that he did not consider the case at all dangerous until a very few days before death.

*Autopsy.*—Considerable emaciation. *Abdomen* distended; upon laying it open the whole of the abdominal viscera were of a uniform color, difficult to describe, perhaps a leaden color tinged with dark green, would be as good a phrase as any other. This color extended to the peritoneum lining the abdominal walls. *Liver* was of a healthy color on the convex side, on the concave was darker than the color of the bowels; an incision into its substance showed a healthy structure, so that its color probably depended upon the shade given to the general color of the bowels by the darker one of the liver. Gall bladder distended by dark green bile *Stomach* very small, apparently healthy, but not opened. Duodenum of good calibre, not opened. Jejunum empty, less colored than the other viscera. Ileum well distended with air, and dark green bile; a portion taken from the upper part of it, showed the mucus coat somewhat injected, otherwise healthy; no part of the ileum, or indeed any of the bowels exhibited any thickening of its coats, or any contraction of its diameter; but upon removing fifteen or eighteen inches of the lower portion of the ileum, emptying it and washing off the bilious matter, several elliptical patches and solitary glands were found slightly ulcerated, without apparent thickening or diminution of the walls. Colon considerably distended. Spleen enlarged, almost globular, very red and much softened, breaking down under the handling, and a considerable quantity of blood was lost from the vessels ruptured in extracting it. The pancreas and kidneys not examined. *Thorax*, heart and lungs healthy.

3d. Sylla, aged forty-five, sick thirty days. Died August 19, 1841.

*Autopsy twenty hours after death.*—About one inch of fat covered the abdominal muscles; omentum well down in the pelvis; stomach moderately distended with air and a little fluid; externally and internally healthy, if we except a small spot just below the central depression, which exhibited a group of innumerable minute red specks, as if the point of a fine needle had been repeatedly applied. The small intestines in various places appeared diminished in volume, otherwise sound until we approached the lower end of the ileum, where externally it was dark and contracted. Upon laying it open, a number of ulcers were to be seen varying in size, some larger than a grain of wheat, others larger than a twenty five cent piece. They extended to the peritoneal coat. In some of the smaller ulcers, the edges were thickened, in the larger there was no thickening of the edges. The intermediate spaces were filled with enlarged veins containing dark blood. Near the ileo-cœcal valve there were some large very irregularly shaped ulcerations. In the cecum and upper part of the colon, were similar ulcers, also near the anus. The colon externally was of a healthy color, but very much diminished in calibre; internally throughout, as dark as a mucus membrane could well be. The rectum in the same condition. In the bowels was a small quantity of semifluid yellowish fecal matter. Some of the mesenteric glands enlarged. There was very little blood in the vena cava or any of the large veins. Indeed there appeared a very considerable scarcity of blood in the cutaneous veins and those in the cavity of the body. *Liver and pancreas* healthy. *Spleen enlarged* and softened. Bladder and uterus apparently healthy externally, but neither was opened.

---

### CHAPTER VI.

### ARTICLE 1.

#### DIAGNOSIS AND NATURE.—GENERAL REMARKS.

"Our diagnosis can never be founded here, as in many other instances, on a few positive physical signs. It must always be rational, not absolute. The evidence upon which our verdict is rendered is wholly circumstantial. Notwithstanding all this, and though cases sometimes occur so enveloped in obscurity as to baffle the skill of the most careful and experienced observers, it is still true, that there are few general diseases the diagnosis of which is so well established, and so certain as that of typhoid fever. * * * Setting aside, as I do for the present, the true typhus fever, there is no disease more readily and positively recognised than a case of well marked typhoid fever, of extreme or even of average severity, when observed from its commencement and followed through its entire course."—*Bartlett on Typhoid and Typhus Fever.*

Just before the quotation first above made, the author informs us that there are no two or three symptoms which show the presence of this disease—that there are no two or three symptoms which may not be absent during the whole course of the disease. He might have made his statement even broader than that. A majority of the symptoms even of those considered most characteristic of the disease may be present and yet the disease absent and *vice versa.*

I shall not go over a repetition of all the symptoms which may be looked for in this disease, but only some of those which I consider of most importance, presuming that the patient is seen during the first week. A tongue whitish, moist, or beginning to become clammy with red papillæ showing through the white coat, and reddish edges and tip; giddiness and tinnitus aurium upon rising; more or less uneasiness of the head; tenderness at the epigastrium or right iliac fossa; tympanitic state of the abdomen, diarrhea, the dejections of a dirty yellowish color, very liquid and very offensive, of a cadaverous odor, with or without griping; great muscular debility; a pulse increased in frequency, it may be increased or diminished in volume and strength; occasionally a cadaverous smell of the body. Although, as before observed we may have a case of typhoid fever with a majority of these symptoms wanting, or a majority of the symptoms without the fever, yet when we have something like the assemblage just enumerated we should be on the look out for the disease. I will offer a few remarks upon the value of each of these symptoms.

The tongue above described is such as I have most frequently seen in recent cases of fevers; but it is also found in cases of intestinal irritation no matter from what cause it may arise. In measles and scarlatina at the commencement, it is very much of the same character.

Giddiness and tinnitus aurium may attend gastric irritation or debility from whatever cause. Tenderness of the abdomen may attend gastritis or enteritis. Tympanites may attend peritonitis, pneumonia, dysentery. Diarrhea, with discharges as described, I at one time considered nearly pathognomonic of typhoid fever, but I have seen it in pneumonia, in measles, and bilious fever and cholera—indeed, during the last few years it has come to be very common in most of our diseases.

Great muscular debility attends many forms of disease in nervous temperaments. With the same amount of accompanying symptoms however, I think the debility in this disease is greater than in any which I have observed. Dr. N. Smith considered a cadaveric smell of the body as pathognomonic; believing that if introduced blindfolded into a room where the disease was, he could detect it. Professor Bartlett seems to be of the same opinion. I will add that during the last summer, a gentleman, whose family had suffered severely in the East with fever, was introduced into the room of a patient, suffering with the disease, who immediately recognised the odor. Still there is frequently some difference between what a man thinks he can do, and what he can actually do. For example: during the winter of 1818–'19, whilst attending a course of lectures in the University of Maryland, my room-mate became sick and had the attendance of Professor Potter, who assured him he had measles. Mr. Sanders insisted that could not be the case, because he had had both regular and French measles. Professor P. however had more confidence in his own diagnosis than in that of his patient, and replied that if he had had forty kinds of measles, the eruption would appear in the next twenty-four hours—that he had no difficulty in detecting the measles by the smell of the patient. Upon his next visit, sure enough, he found the eruption—not of measles as he acknowledged, but of typhus fever. And if I could depend upon my recollection for twenty-eight years, I would say that eruption corresponded precisely with my idea of the rose spots of typhoid fever, and the only spots I have ever seen which did.

By the way, I remember distinctly his teaching that in typhus fever there was always inflammation of the bowels, but I do not remember that he located that inflammation in the ileum; or that he taught that ulceration was common. On the contrary I have always been under the impression that he and Broussais, both taught that the inflammation was in the stomach and duodenum. How true my impression of either case is, I do not know, or whether my notion is not founded on a somewhat natural interpretation of the term gastro-enteritis.

But to return from this digression, I have observed this odor in many patients, ill of typhoid fever—in many, I have not detected it. I have also noticed it in patients laboring under various other diseases.

---

ARTICLE 2.

DIAGNOSIS FROM BILIOUS FEVER.

A person who has been in the habit of treating remittent fever will, upon seeing a case of typhoid fever, readily perceive that there is something of an unusual appearance in it. He may not suspect it to be a fever of a different type, and may have difficulty in ascertaining what causes operate to produce the anomalous train of symptoms. He may even have some difficulty in identifying the anomalous characters, but still he is sensible that the case is one varying materially from such cases as he has been in the habit of seeing. It is true that there are cases which, appearing to partake of the nature of remittent bilious fever and of typhoid fever, may be classed with difficulty; such cases occur respecting most diseases at all analogous to each other; but in the vast majority of cases there can be little difficulty in determining in what list to place a given case. The first distinguishing feature which I shall notice is the period of prevalence.

1. Typhoid fever occurs at all seasons indiscriminately. It is true that in some years it will be found to prevail mostly during the fall or summer; but in other years its period of prevalence will be winter or spring; and very generally it will rage with much the same intensity throughout the year. Bilious remittent fever on the other hand may show, it is true, an occasional case in the winter or spring, but rarely if ever prevails extensively at either period, but rages with violence more or less marked, in July, August, September, October, and November.

2. A second distinguishing feature between these two diseases is the liability to return—relapses. Relapses rarely take place in typhoid fever. By relapse I mean a falling into a state essentially the same as that which existed in the original attack, *e. g.*—if during convalescence from measles, a patient is exposed to cold, and catarrhal symptoms attended with fever come on, I do not call this a relapse; but if during convalescence from bilious remittent fever, a patient from any cause, has a return of his paroxysms of fever, either in the remittent or intermittent form, that I call a relapse; because although there may be great difference in the severity of the paroxysms and of the intervals of return, or even of the trains of symptoms which constitute the paroxysm, yet the latter attack is essentially the same as the first. In this sense a relapse rarely takes place in typhoid fever. On the other hand this disposition is one of the most strongly marked characteristics of remittent fever. Indeed a person who has had intermittent or remittent fever, cannot be pronounced free from a liability to relapse, for a year afterwards.

3. In the cold stage of the two fevers there is a marked difference. In a large majority of cases of typhoid fever, the cold stage it not very distinctly marked; a chilly sensation of greater or less duration being somewhat common, while many cases are not attended by any chill which has fixed the attention of the patient. It is true, that in many cases of remittent fever, there is but a moderate chill at the onset; this chill in such cases being about as severe as those occurring with most severity in typhoid fever; but the access of bilious fever is usually marked by a severe rigor, amounting to decided shivering and chattering of teeth, which it is impossible for the patient to control. This chill, rigor, or ague, is less and less distinctly marked as the disease progresses until it becomes scarcely appreciable.

4. There is a very marked difference in the manner of recurrence of the cold stage. When that stage is repeated in typhoid fever, it is at uncertain intervals and rarely amounting to a distinct rigor, but rather an indefinite sensation of discomfort. In remittent fever, the rigors return with astonishing regularity. It may be that the paroxysm returns every day, or it may be every other day, or at some other interval; but whatever that interval is, it can be soon ascertained. Again, it may return at the same hour at every revolution; or it may return an hour sooner, or an hour later at each period. Having these elements of computation, we are enabled, after carefully noting two or three paroxysms, to predict within fifteen minutes, the time at which the next paroxysm will appear.

5. In typhoid fever, the hot stage comes on very gradually, and the heat of skin, fullness and frequency of pulse are rarely very great in the first days of disease; subsequently the heat of skin abates, but the frequency of pulse increases. In bilious fever the transition from the cold stage to the hot is abrupt, the pulse becoming full and bounding and the heat of the skin great.

6. There is no regular sweating stage in typhoid fever. It is true that the skin occasionally becomes more or less moist, nay there is sometimes a real perspiration, but the occasions upon which this last is observed are rare, and no one can calculate upon the time when it may be expected. In remittent fever, especially during the first days, there is usually a distinct moisture appearing a short time after the febrile paroxysm begins to abate.

7. The tongue at the onset of typhoid fever is usually little different from its healthy state; it gradually becomes red at the tip and sides, a whitish fur forms on the upper surface, through which red papillæ show themselves. As the disease advances it becomes dry, occasionally casting off the fur, and assuming a smooth shining appearance; frequently it has a dark brown coat. In bilious fever, the tongue is usually covered with a whitish fur at the commencement, if not, it becomes so speedily, the fur changing to a tawny or yellow, and in bad cases to dark brown, when it likewise becomes dry. When the tongue begins to cast off its fur, it is done gradually, commencing at the tip, and is always hailed as an evidence that improvement has commenced; the part from which the fur has separated looks well, neither unnaturally red nor dry.

8. The stomach is rarely much troubled with nausea or vomiting in typhoid fever. In bilious fever, gastric derangement with vomiting of bile is a frequent symptom.

9. In typhoid fever diarrhea, more or less considerable, generally attends from the commencement of the attack, frequently it is observed to have existed prior to it. The dejections are different in color and consistence; in a majority of instances not showing a very marked alteration in the quality or quantity of bile, as compared with stools of similar consistence, occurring in persons otherwise healthy; but whatever the color, they are attended by a horrible fetor, which has been termed cadaveric. In bilious fever, there is generally a decided tendency to costiveness, which usually remains more or less marked during the attack. When stools are procured by purgatives they many times have a very offensive odor, but the odor is of a bilious, not of a cadaveric character. It is true that in some epidemics of bilious fever, there is considerable *bilious diarrhea*, the dejections being so acrid as to excoriate the anus.

**10.** There would seem to be more epigastric tenderness in typhoid fever; there is also a notable tenderness in the right iliac region which I have not observed in bilious fever. Tympanites is almost always present to a greater or less degree in typhoid fever; not commonly so in bilious fever.

**11.** It would seem from the observations of others, (not from my own,) that rose colored lenticular spots on the skin are very generally to be found in typhoid fever. I have found sudamina present in something like a third of the cases in that disease. I have also seen desquamation of the cuticle in about the same proportion. I have had a good deal to do with bilious fever, and am not aware that there is any peculiar eruption attending it. It is true that occasionally an eruption appears in consequence of irritation of the primæ viæ; but such eruption varies very much in its appearance. Whelks as large as the finger nail, red, decidedly elevated and attended with a distressing itching; and numerous vibices, red and slightly elevated, have been perhaps the most frequent. The rose spots, desquamation of the skin or sudamina I have not seen. When an eruption appears at all (unless it is petechial) it is commonly at the commencement.

12. The duration of typhoid fever upon an average, may be put down at three or four weeks. Unless in some cases which appeared to be this fever, and which were relieved in a few days, I do not know that I have seen a case decidedly convalescent before the tenth day. I should say that the average duration of bilious fever is not more than two weeks, if that long. Many cases are cured in three or four days, and the ninth day is popularly considered *the* critical day. Either disease may extend to sixty days or more.

13. Another distinguishing mark may be stated, to-wit: the effects of remedies. In typhoid fever general *blood-letting* rarely is called for, and in my observation when used, has been followed by no benefit. In bilious fever, it is frequently of much advantage; directly, in allaying febrile excitement; indirectly, in favoring the operation of cathartics. *Emetics* need to be watched in typhoid fever to prevent their increasing the diarrhea. In bilious fever, they do not produce diarrhea, on the contrary, we frequently give a purgative on the same evening after an emetic—indeed not unfrequently combine them. *Cathartics*, particularly calomel, operate differently in the two diseases. In typhoid fever, a moderate dose is beneficial at the beginning, it may produce bilious purging, but almost always the color is yellow, and the consistence thin; neither can we make much alteration in the quality of the dejections, at least for the better, by increasing the dose. In bilious fever calomel is emphatically *the* purgative upon which chief reliance is to be placed. A moderate dose, say five to ten grains, may cause watery purging, but increasing the dose to fifteen, twenty, or thirty grains changes the character of the dejections very much. They now become consistent, bilious, and dark bottle green in color. Such stools in bilious fever almost universally, (if I were to say universally I think I should tell the truth according to my observation,) are followed by amendment. Such stools in typhoid fever I have never seen from any sized dose of calomel, and I have given it in doses of five to sixty grains.

In some of the cases of typhoid fever in which the stools approximated nearest to that character, the disease continued to grow worse and eventually proved fatal.

14. Typhoid fever does not admit of much activity of treatment. Bilious fever requires considerable activity.

It will not be supposed that the foregoing remarks apply to extreme or irregular cases of disease, but the usual form and as generally applicable to the first week of disease, (unless a later period is obviously referred to,) when we may be presumed to be required to identify the disease.

In the latter stage of disease, that which heretofore has been called the *typhoid stage* of disease, the prominent symptoms will be very much the same, whether that disease be a fever properly so called, (bilious, typhoid, typhus,) an exanthema, on a phlegmasia. So we will say nothing as to that stage.

Since writing this diagnosis I have examined Professor Wood's work on Practice in which he expresses the same opinion as to the difficulty of diagnosis in some cases between typhoid and bilious fevers. "I have seen a case of this kind in consultation, which was considered by a skilful and experienced physician as bilious fever, but which I suspected to be enteric fever, from the state of the bowels, connected with commencing meteorism and the character of the cerebral symptoms. The patient died in about a week, and glandular patches of Peyer were found enormously enlarged," v. 1, p 323. Again, "I believe that occasionally the two complaints are commingled, in consequence of the co-operation of their causes. Thus cases having all the essential characters of enteric fever occasionally end in intermittent; and bilious fevers or affections which cannot be distinguished from them, sometimes show the signs of enteric fever during their progress." p. 329.

---

### ARTICLE 3.

### THE DIAGNOSIS OF TYPHOID FROM TYPHUS FEVER

Is held by those who consider these as distinct diseases to be a matter of no great difficulty. Absence of diarrhea, of tympanites, of tenderness of the abdomen; presence of petechiæ are the marks of typhus. Diarrhea, tympanites, tenderness of the abdomen, gurgling upon pressure in the right iliac region, rose spots, are the diagnostic marks of typhoid.

In order to put this matter in a clear light and show the strength of conviction abiding with many as to the distinction between the two diseases, I will insert a letter from Dr. Power, of Baltimore, to Dr. Bartlett, found at page 272 of his Treatise on Fevers. "The questions you ask have interested me very deeply, and this summer for the first time, I have had ample opportunity to fix definitely my own opinions. What I write you now resumes the opinions also of Dr. Chew and T. Buckler, who saw the disease throughout its whole visitation in this city; nor do I know of one gentleman who had any opportunity of studying it here who differs from us.

"We have had for the last fourteen months an epidemic of typhoid fever in Baltimore. The wards of the almshouse and infirmary have constantly contained a large number of cases of this disease, presenting nothing remarkable, save that the cases had, as a general rule, more the adynamic type than in former years, and required more stimulation. Early in May two vessels arrived bringing Irish emigrants, one from Liverpool, the other from the South of Ireland. Other vessels succeeded these, so that upwards of two hundred cases were treated at the infirmary, and upwards of eighty at the almshouse. These cases were *typhus* so exactly corresponding with Gerhard's description of the Philadelphia epidemic of 1836, that I am constrained to say, I know of no better portraiture of any disease, than that which he has given of typhus fever. I made or assisted at twenty-six post-mortem examinations, in *not one* did I see any trace of the peculiar lesion. In nine of those who died having loose bowels during life, we found either the lesions of dysentery or of diffused muco-enteritis; no mesenteric alteration in any case. The parenchymatous organs and mesenteric vessels were congested with dark fluid blood; and the condition of the spleen, bronchial mucous membrane, lungs and brain resembled what we find in congestive remittent or typoid cases. The stomach was uniformly more altered, and presented deeper traces of inflammation than in typhoid fever. We had fourteen autopsies of typhoid fever during the same period, and it thrice occurred that we had the bodies opened side by side for the sake of comparison made at the time.

"The mode of access, facies, march, eruption, symptoms, treatment, and convalescence are all different between the two diseases. We had both forms of fever at once under our observation, German emigrants and domestic patients, with typhoid; and Irish and English with typhus. Nay, more than this, four of the seamen of the *Rio Grande*, a vessel which brought seventy cases of typhus, had true typhoid fever, and several of the steerage passengers had the same disease. There was no mistake in the diagnosis in any case where the disease was fatal, as proved by the autopsy; and in the successful cases, the difference of the eruption, diarrhea, and meteorism, the peculiar nervous symptoms, the great emaciation, bed sores which were so rare in the worst cases of typhus, that I saw but two, made the diagnosis simple to every clever student. The effect of full stimulant treatment made the difference still more obvious. In short we have here in Baltimore *no doubt*, but the fullest conviction of the non-identity of the two diseases.

"Furthermore, there is the undoubted contagiousness of typhus. Two of the Sisters of Charity at the infirmary—one of whom died—and three out of five resident students took the disease. Four of the hospital assistants, and several of the inmates of the almshouse were attacked; many cases occurred also in the city, where direct intercourse could be detected and proved.

"Here is another interesting order of facts: a German had *typhoid fever* and was eight days in bed under Dr. Buckler, he recovered, and was acting as hospital assistant; in tending the sick immigrants he was seized with *typhus*. Two years ago, Dr. Berryman had, at the almshouse a severe attack of *typhoid fever*. He was appointed resident physician at the new quarantine hospital, where all cases are now sent. He took the fever and died last week—the most promising young man I have ever known, and whose loss has filled us all with grief. Again, one of the immigrants who came near dying in May last with *typhus*, is at this very moment at the point of death with *typhoid fever*.

"There is as much difference in my mind between the two diseases as there is between measles and scarlatina. Huxham has beautifully drawn the distinction between the slow, nervous, and malignant fevers. Excepting the new lights we have in pathology, we can add but little to what he has said. Corrigan, in Dublin, sees the difference between what he calls *enteric* and *typhus fevers*. Dr. Wood, in his late work, appears to me to have handled this subject better than any other of our systematic writers. I perfectly agree with him in his conclusions. It appears to me that we are better placed than either the French or English to study this question without prejudice, and more likely to arrive at the truth." Drs. Gerhard and Wood, of Philadelphia, are equally confident of the correctness of this view of the subject.

If this subject could be settled by the authority of a very considerable number of highly eminent men, it might be now considered as put at rest. But whilst expressing my unfeigned and very high respect for, and confidence in these gentlemen, I am not yet prepared to consider the matter as placed beyond controversy. They prove conclusively that according to their observations, a certain train of symptoms has almost always, (always if you please,) been followed by ulceration of Peyers' plates—that after another train of symptoms, these plates have never been found ulcerated. Hence, they conclude that these two trains of symptoms denote two diseases specifically different. They may be right, but are they necessarily so? According to my observation, and that of physicians immediately in this vicinity, the rose spot does not appear as a symptom of typhoid fever. Am I to set this against the observation of the profession, and conclude that this eruption is not characteristic of the fever? Assuredly not But the symptoms of typhoid and typhus fever do not differ more than those which attend different epidemics of bilious fever. For example, we may have in one summer, an eruption of red pimples or blotches, great nausea and vomiting, and bilious diarrhea to attend it. Perhaps the next summer it will be without either, and obstinate costiveness be present. Yet we hold the two years to have produced the same disease, very much modified, it is true, in its appearance. Again, is there more difference between the symptoms of typhus and typhoid fevers, than between those of the ordinary intermittent, and aggravated forms of bilious fever of the remittent or congestive type? Yet we consider them essentially the same diseases.

Dr. Power informs us that the vessel Rio Grande brought seventy cases of *typhus*, and that four of the seamen and several steerage passengers had *typhoid* fever. Professor Drake found among the immigrants at Gross Island, patients with typhus and typhoid fever: i. e. those with rose spots and those with petechiæ—a rather equivocal evidences of the non-identity of the two diseases; yet certainly not proving their identity. But we find others as well situated to observe, and as well qualified to judge, view the matter in a light very different.

Thus M. H. Landouzy gives in the *Archives Generales de Medecine* for January and February, 1842, an account of an epidemic fever which prevailed at Rheims in 1839 and 1840. If it were possible to identify a disease by symptoms, it would seem that this was unequivocally one of typhus fever. A copious extract of an account of this epidemic, which is too long to quote, is to be found in Bartlett's work on typhoid and typhus fevers, p. 302 *et seq*: "In the six autopsies which were made, the intestinal lesions characteristic of typhoid fever were present. The elliptical plates were either thickened and ulcerated, or they were the seats of ulcerations, and the mesenteric glands corresponding to them were enlarged." In view of this state of things—the symptoms of typhus and lesions of typhoid—Professor Bartlett asks: "Is it possible, that even admitting the two diseases to be essentially dissimilar, under certain circumstances, the causes of both may be so commingled as to give rise to a mixed disease, in which there is a mixture of the elements of both?" *loc cit.* Professor Wood gives into this idea of the occasional amalgamation of the two diseases. Thus, vol. 1, p. 346 of his Practice, he says: "The peculiar disease of the glands of Peyer is never found in typhus, or so seldom as to lead to the suspicion of some intermixture of the diseases, when it does occur." Again—"Nevertheless there are cases which cannot be clearly distinguished; and, as before suggested, it is highly probable that the two diseases now and then exist conjointly together" *Id.*

"Gerhard acknowledges to have met with six cases of true typhus in which the intestinal lesion resembled dothinenterite, and admits that the follicular ulceration occurs in the British epidemic, which he regards as true typhus, but says that it is merely accidental—a complication, not in the ordinary course of the disease." *Dickson's Practice*, v. 1, p. 412. With regard to the amalgation of specific diseases thus suggested by Profs. Wood and Bartlett, it would certainly ill become me to deny the possibility; but inasmuch as the explanation seems to me gratuitous, and so far as I know unsupported by analogy, but rather contradicted by it, I think it ought to be entertained with caution and hesitation. Under such admissions by Bartlett, Wood, and Gerhard, I do not know that it would be too much to say, in the words of *Gualtier de Claubry*, that "there are no means of distinguishing typhus from typhoid fever, in relation either to lesions or the symptoms of the two diseases." Again: "The identity of the two diseases is henceforth put beyond a doubt." *Bartlett*, p. 302.

Of myself, I ought here to say that I know nothing of typhus fever if it differs from typhoid, as I have never seen a case; and all I have said is based upon the writings of others.

---

### ARTICLE 4.

### NATURE.

SEC. 1. *Rose Spots.*—What is the nature of this affection? Is it an idiopathic, contagious, eruptive, self-limited fever?

Or is it a variety of typhus fever? Or is it an element which may enter and assist in making up various diseases? The weight of authority seems at present to be, that it is an eruptive, essential, self-limited fever, whose presence is determined, at least in fatal cases, by the state of the elliptical patches of the lower portion of the ileum. One of the most common and characteristic symptoms being a rose colored eruption on the skin, showing itself during the second week of the disease. It is true, it is not asserted that this eruption shows itself in every case. Louis detected it in forty-nine of fifty-four cases, or seven in eight. Bartlett says that it is present in nine-tenths of the cases. On the contrary, 'of seventy cases referred to by Chomel and Genest, they were absent in sixteen, or about one-fourth'—Wood, v. 1. p. 320. That this is true of the disease as observed by these gentlemen, I am perfectly satisfied; but I am as fully satisfied that such is not true of it as it has appeared in this neighborhood for the last six years. I am assured of this, because I have been much interested in investigating the disease. As before stated, I have examined patients daily during the whole course of disease without finding a single spot: I will add, too, that I have not confined my examinations to a few patients or to a short period. It is my custom to examine all whites. I have also inquired of many of my medical friends, and Dr. Evans, as before noted. is the only one who has seen the eruption, unless Dr. Gano may be an exception. The result of his recollection is given in the former part of this essay.

These facts lead me to doubt whether this eruption is peculiar to this disease—an essential symptom. It may be alleged that fevers purely eruptive, sometimes run their course without their characteristic eruptions making their appearance. This may be true. But is it true that any eruptive fever ever prevailed, in which one-fourth or even one-tenth of all those affected failed to exhibit the eruption? Or is there any instance in which an eruptive fever prevailed for six years in any one location, without the eruption being observed, though diligently sought for? One of the cases, the dissections of which are detailed in this essay, was a white man, who had been subjected to daily inspection by myself during my attendance, which commenced on the fourth day. The eruption was certainly absent in this case; but the intestinal lesion was present. The one then may appear without being preceded by the other. The time too, at which the eruption ought to have appeared had passed before death. Again, sudamina, which are represented as appearing later than rose spots, did appear. The other cases were negroes, from which nothing can be inferred, as I am not aware that the eruption has ever been detected in one of that race.

The space of time during which this eruption makes its first appearance is rather too long for a specific eruption—that is, if we are to reason on this disease from analogy. This period is stated by Louis (on Fever, v. 2, p. 198, Bowditch's editin) to be from the sixth to the thirty-fifth day [thirty-third, B.] according to my computation it should have been the twenty-fourth day. See v. 1, p. 93. Without laying any undue stress upon this, as the observations upon this point, at least so far as I am acquainted with them, are not sufficiently numerous and precise to be of much value, I would remark, that I do not know of any exanthem in which so much latitude in the period of eruption is allowed, there being nothing to interfere with its regular development. By the by, is there an oversight or misprint in the following sentence? 'But there is good ground, I think, for believing that those bounds [of the range of typhoid fever] are wider than those which circumscribe the prevalence of any other strictly idiopathic non-eruptive fever.' Bartlett, p. 85.

I certainly have been under the impression that Prof. B. considered this disease an exanthem.

But as the eruptions are considered as of much importance in distinguishing typhoid from typhus fever, they ought themselves to be distinguishable. Let us examine a lot of cases investigated by Dr. Shattuck, with particular reference to this subject. Of six cases given by him, the 3d, 5th, and 6th exhibited both eruptions. *Bartlett*, p. 294. We will also hear Dr. Stewart upon the eruption of typhus fever. Prof. Bartlett tells us, *op. cit.* p. 210, "Dr. Stewart, amongst others, has within a few years studied with great care and particularity the characteristic appearances of this eruption. He says that the rash is permanent; that is, that it does not consist of a successive eruption of spots; that in all cases it presents the two periods, longer or shorter, of increase and decline; and that in the more severe cases it may exhibit, during the period of increase, four different states, being florid, dark, livid, and petechial. When the hue of the eruption is florid, it disappears readily under the finger: when dark, it still disappears, but more slowly: when livid, semi-petechial or pseudo petechial, as it has been called, it is only partially effaced: and when petechial it is not at all affected by pressure. In many cases it remains florid throughout; in others it presents one or more, and in not a few all these alterations: and after it has reached its height, the process is inverted, and it passes through the various phases of lividity, darkness, redness, and paleness before evanescence. This change from rose spot to petechia was also observed by Prof. Drake, at Gross Island. *West. Journ, Med. and Surg.*, Oct. 1847. *Bost. Med. and Surg. Journ.*, v. 37, No. 8. Also by Dr. Upham, at Deer Island Hospital. *Bost. Med. and Surg. Jour.*, v. 37, Nos. 25 and 26, and early Nos of v. 38.

Respecting this eruption, there is another point not altogether unworthy of consideration. Prof. Bartlett refers, p. 108, to several cases in which this rose colored eruption appeared during a relapse after an attack of this fever* This, it seems to me, is proving too much. I do not know that the eruption peculiar to small pox, measles, or any other exanthem has been observed to reappear after having disappeared in the regular course of disease. If my notion on this point is correct, reasoning by analogy, we shonld not expect the eruption of typhoid fever to reappear, considering it an eruptive disease. If, then, the eruption in the second instance is accidental—not proper to the disease, the same eruption appearing in the first instance, may be accidental—not essential to the disease, but dependent on intestinal irritation.

Here I feel disposed to refer to a case of scarlet fever which occurred to me in December, 1838, as being somewhat analogous to the account given above from Stewart.

"Helen Johnson, aged 2 years, has had no opportunity of contracting scarlet fever, except that her mother has visited a family affected with it—was taken on the 17th December, with an eruption in distinct, elevated acuminated points, not colored; tongue whitish with red papillæ; heat moderate, and pulse as is common in ordinary mild fever. 19th, much the same, except that there has been considerable nausea and some vomiting; tongue cleaning off at the tip and edges, rather smooth, but neither red nor dry. 21st. Has been quite lively since last report; ate heartily yesterday. At present feet very much swelled; eruption on feet and legs distinct, red, from one to three lines in diameter; also a considerable patch on the thigh; tongue clean, smooth, papillæ distinct but not red, moist and nearly natural in color; considerable hoarseness and soreness of flesh. 22d. Feet less swelled, eruption on feet, legs, and thigh purplish and fading, tongue

* A similar case is noticed in this Essay.

as yesterday, child lively. 28th. The red blotches are still apparent on various parts of the body; appetite good, tongue assuming the natural appearance." I think it not a little singular that the mother of this child sickened on the 20th of same month, had the same elevated acuminated colorless eruption upon the hands and arms; the same red spots upon the body; had the skin exfoliating on the 28th, that of the hands coming off in patches of two or three inches in diameter. I will add that this lady died in 1841, of perforation of the bowel, occuring in typhoid fever."

After considering maturely the above extracts from Stewart; after noticing the observations of Drake, at Gross Island, and Upham, at Deer Island; after having seen a case apparently parallel in scarlet fever, is it unfair to doubt the distinction between the rose spot and petechia? If the same identical spot is a rose spot one day, a petechia the second, and a rose spot again on the third, it does seem to me that distinguishing them apart is alike impossible and useless. Hence we must not be surprised—nay, we may expect that the rose spot and petechia shall eventually turn out to be the same eruption under different modifications.

SEC. 2. *Sudamina.*—Louis found sudamina present in about two-thirds of his cases. This eruption is said to be found more frequently in this than in any other acute disease. It has been found in a large proportion of cases in this vicinity. Here, then, is a second cutaneous eruption, more or less characteristic of typhoid fever. Indeed Dr. Hale, of Boston, who had good opportunity of studying the disease, enumerates both the rose spot and sudamina as among ' the distinctive marks of typhoid fever." *Am. Jour. Med. Sci,* v. 25, p. 396. I do not know of any other exanthem being allowed more than one cutaneous eruption. Is typhoid fever a disease which bids defiance to analogy?

Sec. 3. *Peyer's Patches.* We come now to the intestinal affection—the dothinenteritis, which I think those who place this fever among the exanthemata ought to consider the characteristic eruption. It is held to be characteristic of the disease—essential to it—that it is found in no other acute disease, if in any at all. That this affection of the intestinal mucous membrane is to be found in this region, dissections have sufficiently shown. It has been found, too, in cases which exhibited a fair proportion of symptoms marking typhoid fever. But whether that condition is the characteristic mark of a specific disease, let us now inquire.

We find that Dr. Lombard, of Geneva, who appears to have been familiar with typhoid fever, both as to its symptoms and pathology, for six years in Germany, upon visiting Great Britain saw cases which he declared could not fail to exhibit ulcerations which he considered essential to that disease. Upon examination of the bodies, however, he was astonished to find no lesions such as he expected.—*Amer. Jour. Med. Sci.*, v. 26, p. 400. *Bartlett*, 257. Dr. Shattuck, of Boston, after studying typhoid fever in Paris under Louis, at his request, examined cases of fever in England, with a view of determining the identity or non-identity of the fever of the two countries. He concludes that typhoid and typhus fevers are distinct, though both prevailing at the same time. Yet he gives us a case of what he considers typhoid fever, which appears indeed to have been as well marked as most cases of the disease; but "there was no appreciable alteration of the elliptical plates or of the mesenteric glands.—*Bartlett*, 294. Fouquier reports a case in which the symptomatology of typhoid fever seems to have been pretty clearly marked, and in which the elliptical plates and mesenteric glands were found almost free from disease.—*Id.*, 116. Prosper Dor reports a case, of which Professor Bartlett says: "Certainly in this case the diagnosis during life would have been sufficiently clear and positive." Yet, "examination after death showed the intestines healthy."—*Id.*, 117.

Having briefly referred to some cases in which the symptoms would have seemed to point out ulcerations, or at least serious disease of the elliptical plates which nevertheless failed to exhibit that lesion upon examination, I will now turn to the converse proposition. Here it will not be amiss to make an extract from Professor Bartlett's work. "In all cases of typhoid fever there is lesion of the small intestines. This lesion is peculiar. It is found in no other disease," p. 58. Of this "invariable and characteristic lesion," I cannot give a better or perhaps a more succinct description than is to be found in the work referred to. "In a small proportion of cases, consisting of those which terminate early, the elliptical plates together with the subcellular muscular tissue are merely increased in thickness, with redness and softening. This increase of thickness is such that the edges of the plates project from one to two or three lines above the surrounding mucous membrane. Sometimes the hypertrophy of the plates and of the subjacent cellular tissue is quite simple, the color and consistence of the membrane remaining unaltered. This simplest form of the lesion, like all others, which are more complete, is universally found most advanced and most strongly marked at the lower extremity of the ileum. Each successive plate as we go upward along the intestinal track from the ileo-cecal valve, is less profoundly altered, till we arrive at those in the natural condition. The number of plates thus changed is very various. [From one or two to thirty or forty.] The surface of the thickened plates frequently presents a granular or finely mamelonated appearance occasioned by an enlargement of the gray orifices of the cryptæ which go to make up the plates. This condition becomes very manifest when the gland is detached from its subjacent tissue and held between the eye and the light. At other times the surface of the thickened membrane corresponding to the plates is quite smooth and level.

"In a great majority of cases, the plates, instead of being merely thickened with or without redness and softening, are more or less extensively the seat of ulcerations. These ulcerations vary very much in size and number. It frequently happens, for instance, that in proceeding from above downwards, in our examination, after having passed over several plates simply thickened, we come to one of them in which there is a single circumscribed ulceration with perpendicular edges, extending more or less deeply into the thickened tissues. As we go on towards the termination of the intestine, the ulcerations become more and more numerous and extensive, till at last for several inches next the valve the plates are entirely destroyed, and we find only ulcerations corresponding to their sizes and shapes occupying their places.

"These intestinal ulcerations are commonly more or less rounded or oval in their shape. Sometimes, however, their borders are irregularly jagged and angular. So their edges are in most instances pretty regularly perpendicular and smooth, but sometimes they are ragged and shreddy. The bottoms of the ulcerations vary of course with their depths. They consist sometimes of the cellular tissue immediately under the mucous membrane; sometimes of the muscular coat; and sometimes of the peritoneal covering. Occasionally this covering itself gives way, perforation takes place, and the contents of the intestine are discharged into the cavity of the peritoneum."

Keeping this description in mind—which indeed I think very excellent, (if we supply a small omission, viz.: that the membrane between these thickened or ulcerated plates, as the case may be, is frequently more or less highly injected, and traversed by enlarged veins filled with dark blood,) let us see whether this state of things exists in no other disease.

Broussais' Chronic Phlegmasiae, vol. 2, p. 106, gives a case of intermittent fever changed into continued fever; death on the sixty-ninth day; in which among other appearances observed upon dissection, we have: "The mucous membrane of the small intestines in its upper part presented some isolated red points; lower down near the end of the ileum it was of a deep red, granular, black, and generally sphacelated and ulcerated. An analogous disposition throughout the whole length of the colon. All the granulations were so many small ulcers with loss of substance of the membrane; the appendages of the intestine studded with small black glands."

It may be objected that this, and some other examples cited in this essay are not the ulcerations marking typhoid fever—that these last consist of inflammation and ulcerations of the solitary and Peyer's glands—that such is the legitimate inference is true—that such is more than intimated by Professor Bartlett is likewise true. He says after Chomel: "That one of the most constant and uniform characteristics of secondary lesions, consisting generally of specific inflammations, is the fact of their being *disseminated;* of their occupying numerous and circumscribed spots in the tissues and organs of the system," p. 142. Again: "Now in every respect, the intestinal lesion of typhoid fever corresponds to this class of pathological alterations. It is disseminated; occupying the same glandular tissue at different points of the intestinal mucous surface," &c., p. 141.

It is true he does not assert that the ulcerations are confined to the glandular structure and the mucous membrane covering it; but such is clearly the impression conveyed by the language. If this interpretation is the correct one, the objection above supposed is valid. But that such is not always the fact I have evidence to me conclusive. In Sylla, the autopsy of whom is given in this essay, many of Peyer's patches and the solitary glands were ulcerated; and for aught I know, the ulcerations may have been confined to the bounds of the glands; but at the lower end of the ileum there were several very irregular ulcerations, one of which was two inches long, by one and three quarters wide; a tongue of membrane not ulcerated, running up the broad end of the ulcer about half an inch wide and three quarters long. In the cecum and colon were similar irregular ulcerations. I hold that these ulcerations were not situated in Peyer's plates at all, because the form and proportions were not such as to admit that interpretation. Or if they were so situated, the contiguous membrane must have been involved. I had the pleasure of showing the casts of some of these ulcerated portions of intestine to Prof. Bartlett, who recognised them as examples of the lesions of typhoid fever.

In *Horner's Path. Anat.*, p. 307: "In a patient dead of phthisis pulmonalis, it is said the jejunum and ileum abounding in large ulcers of the mucus coat, which went around the circuit of the gut and were twelve or eighteen lines wide. * * * The beginning of the colon, on internal face, covered for some five or six inches by ulcerations, also the adjoining part of the ileum." Again, of a patient dead of tubercular consumption, supervening upon epilepsy it is said: "The glands of the mesentery were enlarged. * * * Numerous patches of red inflammation, looking like the precursors of ulcers existed in the mucous coat of the intestines. There were several ulcers from two to five lines in diameter, in the small intestines, and a few in the large," p. 370.

In Wistar's Anatomy, by Pancoast, these glands are represented as very frequently the seat of lesion in phthisis as well as in typhoid fever; no attempt to discriminate between them, being made.

In connection with these alterations as observed in phthisis, I will introduce an account of a case which I saw last spring, (1847,) which I consider of some value.

Matthew, aged twenty-two, until a year ago remarkably stout and healthy. Then became affected with severe cold in consequence of exposure. He continued more readily influenced by fatigue than common, yet not to such a degree as to attract marked attention. About Christmas, he was seized with hemorrhage from the lungs, he coughed much, and was disabled from work. He, however, improved afterwards, and thought himself able to break hemp. He was soon obliged to desist on account of recurrence of hemorrhage. March 5.—I saw him rather accidentally; at which time his pulse was ninety; frequent hacking cough, but no blood expectorated; skin ashy and dry; tongue furred white; muscular weakness; respiration frequent and easily hurried; considerable emaciation. He took about this time, some doses of calomel, opium, and ipecac; and subsequently some small doses of ipecac and tar water. He seemed to improve for a time, and about the first of April drove an ox-cart to town, a distance of five miles, without much fatigue. He then appeared better, but his pulse remained frequent. On the 13th of May I was desired to see him, as he was thought to have typhoid fever, and his master thought that, possibly if he could survive, the attack might effect an improvement in his condition. I found him covered with a profuse perspiration, tongue furred white; cough as before, but discharging considerable quantities of pus; respiration very quick, considerable dullness on the right side of chest, left tolerably clear; no diarrhea, but some tympanites; pulse, sitting, one hundred and thirty-five; lying, one hundred and twenty; slightly delirious. I gave him small doses of blue mass and Dover's powder, directed attention to bowels and general comfort; and informed his master that I saw nothing but the progress of phthisis, that—I thought the case hopeless. I did not see him again. June 2.—His master informed me that he had died that morning, and as I had expressed a wish to examine his body, I could do so. He also told me that Matthew had continued to grow weaker from the time I saw him. His pulse smaller; his mind more unsteady; until a few days before death he was compelled to lie in bed altogether—having considerable trembling of the hands—pulse too frequent and weak to be counted in his tremulous condition. He had continued to cough, but about a week before death ceased to spit pus—had complained of no pain or tenderness in abdomen—had continued costive, for which a laxative had been administered every alternate day until the last four days of his life, during which he had no stool.

*Autopsy seven hours after death.*—Emaciation very considerable but not extreme. Abdomen not distended. *Thorax.*—Heart natural; left lung generally healthy, but a few tubercular masses interspersed through its substance; right lung a complete mass of tubercular aggregations, with the intervening parenchymatous tissue condensed and reddened; the whole lung attached to the pleura-costalis by adhesions apparently not of very long standing. *Abdomen.*—Stomach apparently healthy externally, not opened; duodenum of good appearance—upper part of jejunum of same description; the lower portion and ileum dark and contracted in diameter, except in small portions here and there, which were of healthy color and dimensions. Along this range at distances of from six inches to a foot, were patches distinctly thickened, as observed by feeling on the outside; upon opening the bowel, they were found exalted in color, projecting about a line above the surrounding surface with minute ulcerations on the surface. These patches were fifteen to eighteen lines long, by eight or ten wide. Many solitary glands were ulcerated. In the last two feet of the ileum were only two of these patches; above that, they were frequent. The appendicula vermiformis was very dark, which color was communicated to that portion of the peritoneum with which it was in contact. There was also a small collection of pus at this point. *Colon* much distended except for two or three inches near the commencement of the descending portion, where it was of less calibre than natural. Upon laying open this portion there was considerable thickening of the mucous coat with slight discoloration, also an ulceration of a line in diameter. Here was a minute particle of well elaborated feces, such as are frequently found in the watery stools of typhoid fever, about the time amendment commences; it was perhaps a fourth of an inch long by a sixteenth. Otherwise the bowels were more completely empty than I ever saw them, or indeed imagined it possible they could become. It was only in portions that the mucous membrane was even tinged with apparently healthy bile. In various other portions of the colon there were spots of discoloration and evident thickening. *Liver.*—On convex surface healthy, on concave rather dark, but apparently healthy in structure. Gall-bladder filled with bile. *Pancreas and kindneys* healthy. *Spleen* nearly globular, dark claret color, with numerous tubercular masses on surface; upon being laid open, it was very thickly studded with tubercular masses, globular, of the size of a small rifle bullet. *Mesenteric glands* slightly if at all enlarged; the largest of the size of a small filbert; it was not colored, and being cut into presented several minute points of tubercular matter. Behind the root of the mesentery were several masses of tubercular deposit. To put this matter in as clear a light, in all its bearings, as possible, I add that when I first saw this boy in March, he was in the room with his brother laboring under typhoid fever.

Was this a case of unmixed phthisis or of typhoid fever supervening on phthisis? Are there any cases on record in which typhoid fever came on in the last stage of

Recently, November, 1847, I saw a case of fever in a patient who was thought to have been afflicted with consumption for some time. I could not detect any pectoriloquism, but some dullness of chest. Although very anxious to make an examination after death, I was not allowed the privilege.

For the purpose of acquiring the most satisfactory information upon the condition of Peyer's plates in typoid fever, as compared with small pox, measles, &c., I requested information of Dr. Holmes, Professor of Anatomy, and Dr. J. B. S. Jackson, Professor of Pathological Anatomy, Boston. I applied to these gentlemen because I believed that this subject has been more thoroughly investigated in New England than in any other part of the United States. Professor Holmes says: "I am not aware that ulceration of Peyer's patches are met with in measles or small pox. The mark of ulceration in phthisis consists in the presence of tubercle; but if none of this substance be present there may be a great deal of doubt—as in a case which I saw some months since in South Boston." Professor Jackson informs me that he has often seen Peyer's patches red in measles, scarlatina, pneumonia, and croup, but never ulcerated. He has never examined them in small pox, and is not aware that they have been found ulcerated in that disease. These remarks apply to observations made at periods when in cases of typhoid fever, ulceration would have been expected to be present. In phthisis he does not think he has seen ulceration of the patches without more or less tubercular deposit about them, though such ulcerations are, he believes, generally found in the large intestines.

I not long since held a conversation with a distinguished gentleman; during which he remarked, that an expert pathological anatomist would readily detect the difference between the ulcerations in phthisis and those of typhoid fever; but he did not feel himself competent to do so with certainty. He mentioned the state of the spleen and mesenteric glands, as being sufficient to determine the point. My reflection at the time was—if you cannot determine the point, I guess its determination must be a matter of conjecture rather than of demonstration; and involuntarily thought of Professor Potter and the measles. Let it be remembered that we are considering this lesion as to its peculiarity in typhoid fever—as being of itself sufficient to demonstrate the existence of that disease, not as it is interpreted by previous history of the case or concomitant lesions of other organs.

Professor Bartlett *op. cit.* p. 119 says: "There is no good reason to think that positive ulceration of the intestinal follicles usually takes place in mild cases, and perhaps not in many of moderate severity." "It is certain that numbers of patches are frequently found without ulceration—that there is a time in every case when none are ulcerated. Sudden congestion or other complication might supervene at this time and cause death." In what would the patches in these causes differ from their appearance in measles, scarlatina, or croup. Again, "There is another peculiar appearance of the diseased plates, which is found in a certain proportion of cases; according to Louis, in somewhat less than one-third. This seems to consist in a morbid change or transformation of the subcutaneous cellular tissue. Instead of being simply hypertrophied, with or without redness or softening, as in the cases already described, this tissue is converted into a substance of a yellowish color, destitute of any traces of organization, presenting a substance somewhat glossy when cut, and about as hard and friable as crude tubercle." How are these cases, which according to Louis amount to nearly one-third of the whole number, to be discriminated from those of phthisis, if we look to these plates alone for the distinguishing features? It does seem to me, that if the description above given, is not one of tubercular matter, it is one which it is very difficult to discriminate by words from it.

There are two points concerning this lesion which it may be proper to mention, more as a means of keeping the course of the disease before the mind, than in any way elucidating its pathology.

1st. That the disease may terminate fatally, notwithstanding some considerable progress may have been made towards restoring the healthy condition of these patches. Thus, we see in Louis, v. 2, p. 166, in a patient dying upon the twenty-first day, some progress in restoration had taken place. This had been a case of considerable severity. Again, at p. 62 we have another autopsy of a patient dying on the twenty-ninth day evincing the same state of progressive improvement, as evenced by a grey, bluish tint in the parts ulcerated, and the mucous membrane generally. Again, v. 1, p. 122, we have a considerable progress towards healing in a patient dying on the forty-third day. One remark in passing: about the twentieth day, according to our present information, seems to be the earliest period at which the process of restoration commences. Here we have death occurring at that period notwithstandidg the appearance of healthy action was visible. I do not think that it is common in eruptive fevers, for the patient to die after the eruption, having progressed regularly, has begun to decline at the shortest period. I have seen cases of typhoid fever, it is true, in which congestion of the lungs or other secondary symptoms supervene suddenly and carried off the patient, he seeming to die, not of the fever but of congestion. But such does not seem to have been the fact in the cases referred to.

2d. There seems to be very great lattitude as to the time this cicatrization commences. We have seen that it may have made some progress on the twenty-first day. On the other hand we have a case, *op. cit.* v. 1, p. 130, of an apparently decided convalescence, setting in on the twenty-first day, continuing for eighteen days; a relapse and death on the sixty-fith day, in which no progress towards restoration in these patches had been made. It would thus seem that there is no certain relation between the period of disease and this lesion; nor between the healing of this lesion, and the life or death of the patient. It may however be said that improvement may have commenced in these patches at the time convalescence appeared to begin. To this there are two objections: 1. In one of the cases quoted, healing had commenced although the case was going on to a fatal termination. 2d. In relapses in eruptive diseases, the eruption does not reappear.

Describing the pathological appearances in patients dead of infantile remittent fever, Mackintosh says: "The ulcerations in the ileum and colon, strictly resemble those which I have afterwards to describe in the bowel complaints of children, except that the whole mucous surface of the colon is occasionally involved in one sheet of ulceration, with a rough and ragged surface and hypertrophy of the other coats, as observed in many cases of phthisis pulmonalis," vol. 1, p. 179. Again, treating of the inflammation of the stomach and bowels he says: "On some occasions we shall see numerous dark-colored distinct points, somewhat elevated with a depression in the centre, which are mucous follicles enlarged; in some places a number of these points will be seen to coalesce, sometimes into a circular space, but in general they are more of an oblong shape. The surface is elevated and sometimes spongy; and upon making a section through this part, it will in general be found that the submucous tissue is principally involved in the disease, and occasionally the muscular tissue also. On looking at the surface through a glass, ulcerations will be discovered. This appearance is most commonly observed in the lower part of the ileum and caput cecum, in children who have died of bowel complaint. Occasionally numerous distinct points will be observed, as if a penful of red ink had been spattered on the surface of the mucous membrane. * * * On other occasions ulcerations are observed of a circular or oval form, with defined edges, attended by loss of substance not only of the mucous membrane and the submucous tissue, but extending into the muscular coat which may be seen in different places in a ragged state. * * * * Occasionally indeed, the ulceration extends through all the tissues allowing the escape of the contents of the bowels into the abdomen. * * * Ulcerations are sometimes circular sometimes oval; sometimes they run in lines, and on other occasions are found to be irregular in shape. In size they vary from that of a millet seed, to be so extensive as to occupy a large space. Sometimes the whole intestine. * * * Ulcerations of the small intestines are for the most part, found in that portion of the tube most distant from the mesentery," p. 337, *et seq.* Again, speaking of *Tabes' Mesenterica* he says: "At other times the lower part of the ileum and cecum are affected; and occasionally ulcerations of the jejunum, increasing in number however in the course of the ileum," p. 353.

In the forty-fifth volume of the Edinburgh Medical and Surgical Journal are some observations on continued fever as it occurs in the Glasgow hospitals, by Dr. Robert Perry, in which he considers the intestinal lesion as an accidental complication of typhus fever, and not less frequently of small pox; and says that in the latter disease, the morbid appearances in the intestine are the same as those which occur in dothinenteritis itself, which disease, he says, may exist as an affection *per se*, characterised by its peculiar symptoms."—*Bartlett*, 296. It is true that Professor B. questions this testimony; but from what I have seen I am by no means certain of its want of truth *e. g.*—in 1845 I had a patient who had been complaining for a week with great muscular debility, occasional chills, frequent, weak pulse, tongue whitish with red papillæ, buzzing in the ears; distended tender abdomen, considerable diarrhea, dejections of dirty yellowish color and cadaverous odor, which in fact for two days I considered a pretty fair case of typhoid fever; but luckily, on the third, the eruption of measles undeceived me. If this man had died, I think it quite probable that the lesions of typhoid fever would have been found; if not ulcerations, that equivocal thickening which could very well have been interpreted to be the genuine affection.

Treating of *painter's colic* Mackintosh says: "The following is an abstract of the appearances found on dissection, in the bodies of a number of individuals who died of this affection in the hospital of Beaujon under the care of M. Renauldin: redness and ulceration of the mucous coat of membrane of the alimentary canal, and often an enlargement of the mesenteric gland corresponding to the inflamed and ulcerated portions of the membrane. The redness varied from that of bright rose even to violet or brown. It was disposed in points, in patches, in streaks, and sometimes occupied an extent of several feet. The thickness was variable. The ulcerations were found almost always towards the termination of the small intestines, near the valve of the colon, which was sometimes destroyed; and in cases where diarrhea prevailed, ulcerations were found in the colon; and sometimes they were observed in the stomach. They were deep and numerous, and sometimes the stomach and intestines were perforated." "Roche and Sanson inform us that M. Renauldin had two hundred and seventy-five cases in the years 1821, '22, and '23."—*Mackintosh's Prac.*, v. 1, p. 291.

"In teething children the glands are affected in precisely the same manner as in typhoid fever. Dr. Hale has seen six cases in his practice, of children who have died during teething in whom the glands were thus affected."—*Am. Journ. Med. Scien.*, v. 25, p. 399. It will be noted, that Dr. Hale is one of those who has contributed most to our knowledge of typhoid fever, and as such, his observations are entitled to much weight.

"Dr. Marizini has written to the Academy of Sciences of France, that he has found the alterations of the intestinal follicles characteristic of typhoid fever, in the body of a seven months child, who died twenty or thirty minutes after birth. Many physicians witnessed the autopsy, and can testify, he adds, to the truth of the statement.—*Amer. Jour. Med. Scien.*, n. s., v. 4, p. 173.

As this disease is to me a subject of great interest, I hope I shall be excused for introducing here, a few notes of cases, which may at first sight seem irrelevant, which however I think not altogether without analogy to our present subject. They are from Abercrombie's Treatise on the Stomach: *Case ninety-first*, from symptoms and appearances on dissection, was pretty clearly one of typhoid fever. *Cases* ninety-third and ninety-fourth were doubtless those of perforation of the bowel occurring in the same disease. *Case* eighty-fifth seems to have been of the same character. At the commencement, after a dose of castor oil, (there being great uneasiness in the abdomen,) he had numerous evacuations consisting mostly of blood. This was on the 8th of October. 9th. Uneasiness of abdomen, chiefly referred to lower portion. Stools still almost entirely of blood. 10. Pulse nearly natural; much pain and tenderness in lower part of abdomen; some dysentery. The evacuations now more abundant in quantity, and remarkably changed in character, being watery, dark colored, and with a remarkable and peculiar fetor." They are compared by Mr. White to the washing of putrid flesh. "After the diarrhea, a regular decline with adynamic symptoms, and died on the 16th." This case seems to have lasted only ten days. Upon inspection, the whole colon, from its caput to the extremity of the rectum, was greatly and uniformly distended. From the anus to the middle of the arch, the intestine was of a uniform dark color, as if gangrenous. From the middle of the arch to the caput coli, the appearance was more healthy, but was variegated by numerous patches of deep red or livid color. The intestines being laid open, the mucous membrane from anus to middle arch, of deep, uniform dark color, soft, and easily separated; muscular coat in same condition; peritoneal coat healthy. From middle arch to cecum, there were elevated irregular patches of a dark red color, with interspersed portions of more healthy color. Towards the caput coli there was an appearance of erosion or superficial ulceration; and on the inner side of the caput coli were several distinctly defined ulcers. The ileum for a few inches from its junction with the caput coli was slightly distended and its mucous membrane reddened. Other parts of the canal healthy." The author remarks: "It may be of importance to state that the wife of this man was affected with this same disease in a very protracted form, from which she had not entirely recovered at the end of two months. Three sons were also successively attacked, of whom one died." *Case* eighty-sixth fatal on the ninth day after a fair set of symptoms indicating typhoid fever. The mucous membrane was healthy in the stomach and in the upper portion of small intestines. In the ileum there began to appear spots of increased vascularity, which were at first at considerable distances, but afterwards became more numerous; and for about twenty-four inches of the lower end of the ileum, the whole mucous membrane was of a uniform deep red color, without any remarkable change in its structure. In the caput coli the same dark, red state of the membrane continued; and it was here covered by numerous well-defined ulcers, some of them of the size of a sixpence. In the ascending colon, there was a more irregular state of disease, consisting of wandering undefined ulceration, variegated with dark fungoid elevations of portions of the mucous membrane. In the arch of the colon the disease assumed a different character; for it there consisted of small well defined ulcers, the size of a split pea or smaller; they were quite distinct from each other, and the mucous membrane between them was of a pale color and quite healthy. In the descending colon, the whole of the mucous membrane showed one continued surface of disease—being of a dark-brown color, fungoid and spongy without any defined ulcerations. *Case* eighty-ninth, dysentery, stools copious, varied very much in appearance, being sometimes slimy, sometimes watery, and sometimes consisting of mucus mixed with green matters of various shades. *Autopsy.*—Some superficial ulcerations towards the lower extremity of the colon, but the principal seat of disease appeared in the caput coli, in which were numerous fungous projections ulcerated upon the surface. In the ileum four inches from its lower extremity, there was a portion in a state of recent inflammation, covered with a false membrane. Small abscesses in the liver; mesenteric glands enlarged.

Here we have a lot of cases, some of which, by symptoms and appearances on dissection were pretty certainly cases of what would now be called typhoid fever. Some in which the symptoms would seem to indicate that to have been the disease, but the dissections did not show the peculiar lesions of typhoid fever; but either a corresponding lesion situated in the colon or an analogous one situated in the ileum. The last is considered dysentery by the author, but it seems doubtful if it was not truly one of typhoid fever. At any rate the appearances on dissection were analogous to those having a striking resemblance to typhoid fever.

Whatever others may think of the light in which I have viewed these cases, I am of opinion that a patient and careful examination of them, and of some others of the same author, with an eye to their analogy to typhoid fever, will amply repay the man who makes it.

But it may be urged against many of these cases, that they are not described with sufficient minuteness to enable us to form a proper opinion as to their value. That English writers do not discriminate between typhus and typhoid fevers. That many cases which have been described as infantile remittent fever, and even cholera infantum, were in truth typhoid fever. In one word that things identical have been considered as altogether dissimilar. To prevent, on the one hand confounding things which are really different, and on the other separating those which are alike, is many times a work of great difficulty. Such is the matter now in controversy.

Let us recapitulate this section somewhat. I have declared that the typhoid eruption, the rose spots, though diligently sought for, had not been found in this vicinity. I have exhibited proof, that in some considerable proportion of cases, say from one-tenth to one-fourth, it is admitted to be absent. That in some cases, (whether of typhoid or typhus fever it matters not,) both these and petechiæ appear upon the same patient. That in some cases they are convertible into each other—running from rose spots to petechiæ and *vice versa.* That the lesions of the elliptical plates have not been found where, from the symptoms, we had a right to expect them. That these lesions have nevertheless been found, where we had no right to expect them. That in other cases we have found analogous lesions in the ileum, and corresponding lesions situated in the colon.

It may be argued that the case quoted from Broussais was one of genuine typhoid fever, occurring after one of intermittent fever; or that it was typhoid fever from the beginning. The history of the case as given does not seem to warrant that conclusion. Still the author may have had his attention so fixed upon some features of the case, as to prevent his seeing others, important to the proper understanding of it.

In the cases from Horner there is evidently no suspicion entertained by the author, that anything of what is usually called fever, affected the patients. Matthew's case, given before, I considered one of pure consumption and there was nothing to change my opinion; and I have the satisfaction of having a confirmation of this opinion in the judgement of Professor Bartlett.

Perhaps it would not be uncharitable to suppose that some of the cases reported as infantile remittent, or even as cholera infantum were in truth those of typhoid fever; as I cannot conceive that it would be difficult to confound them; but I should suppose painter's colic ought not to be so confounded.

Upon a careful examination of the whole ground, shall we conclnde that the lesions of Peyer's plates are invariable and characteristic of typhoid fever—that they are to be found in no other disease? Truly I cannot bring myself to do so. Shall we conclude that they do not indicate a distinct form of disease or even a distinct disease? I am not prepared for that either. Shall we say that they are an accidental symptom which may occur in many different diseases, and even exist independent of other symptoms? It behooves us to be cautious in this matter. In our profession we have had a sufficiency of hasty conclusions, drawn from insufficient observations, which have been replaced by other conclusions drawn from facts equally unsatisfactory. I hope we shall leave this matter under investigation, until we shall have accumulated facts sufficient to enable us to come to conclusions, which shall remain permanent.

*(To be continued.)*

